# Drawing transmission graphs for COVID-19 in the perspective of network science

**DOI:** 10.1017/S0950268820002654

**Published:** 2020-11-04

**Authors:** N. Gürsakal, B. Batmaz, G. Aktuna

**Affiliations:** 1 Faculty of Economics and Administrative Sciences, Fenerbahçe University, Istanbul, Turkey; 2 Open Education Faculty, Anadolu University, Eskisehir, Turkey; 3 Public Health Institute, Hacettepe University, Ankara, Turkey

**Keywords:** COVID-19, network science, reproduction number, super-spreader, transmission graphs

## Abstract

When we consider a probability distribution about how many COVID-19-infected people will transmit the disease, two points become important. First, there could be super-spreaders in these distributions/networks and second, the Pareto principle could be valid in these distributions/networks regarding estimation that 20% of cases were responsible for 80% of local transmission. When we accept that these two points are valid, the distribution of transmission becomes a discrete Pareto distribution, which is a kind of power law. Having such a transmission distribution, then we can simulate COVID-19 networks and find super-spreaders using the centricity measurements in these networks. In this research, in the first we transformed a transmission distribution of statistics and epidemiology into a transmission network of network science and second we try to determine who the super-spreaders are by using this network and eigenvalue centrality measure. We underline that determination of transmission probability distribution is a very important point in the analysis of the epidemic and determining the precautions to be taken.

## Introduction

The first half of 2020 passed with the whole world dealing with the COVID-19 outbreak. First, many countries implemented lockdown, and then reopening came to the agenda. However, at the time of writing this paper, there was an important increase in the number of infections all over the world and we would probably spend the second half of the year dealing with the COVID-19 issue and lockdowns again. The fact that COVID-19 is a relatively new virus also challenges scientists and scientific analysis have to navigate the uncharted territories [1].

This paper attempts to establish a link between the fields of statistics, network science and epidemiology using an interdisciplinary approach. From a micro-point of view, this connection, which was tried to be established, was made by converting transmission distribution of statistics and epidemiology into a transmission network of network science. From a micro-point of view, the study also tries to contribute to efforts to stop the epidemic by identifying who the super-spreaders are, and then by researching and identifying their various characteristics.

COVID-19-positive people who have strong social connections and do not consider social distancing are potential super-spreaders. Of course, not everyone included in this definition will be super-spreaders, and this definition will only include potentially super-spreaders. In such a case, let us assume that we are at zero point in time, before the pandemic has started.

Such a social network will show us that no one is infected yet, but who is disregarding social distance, and who has strong social connections, both ignoring social distance. This type of social network will give us clues about who could be potentially infectious and who might be super-spreaders. We can call this network the pre-pandemic network, as well as the ‘pre-analysis network’ at any time in the pandemic. During the pandemic, we can create a second ‘pandemic network’ later than when we got the pre-analysis network, this time to give us social connections of the infected, the non-infected and the super-spreaders.

As a result, we will have two networks: the first one is the ‘pre-pandemic’ or ‘pre-analysis’ network and the second one is the ‘pandemic network’. It is possible to think of the first of these two networks as a funnel. Also, people who are not potentially viewed as super-spreaders in our first network may become super-spreaders over time. A detailed comparison of these two networks will teach us a lot. Even if it seems surprising and futuristic at first, this kind of pro activity can be used to determine which people will be super-spreaders using networks.

In an age when people can't live without cell phones, forming both of these networks will not be too difficult for authorities. It is now a known fact that it is not difficult to see who is close to whom with the signals emitted by mobile phones. Although at first it seems that personal privacy will prevent the acquisition of such networks, we know that this is not a problem in some countries such as China.

‘Mobile location data provides a granular solution for consumer understanding. Combining this understanding with other datasets are helping to solve business problems and achieve goals across many different industries’. The sad and tragically funny thing is that although such data are available for consumer estimates, they are not used in the case of COVID-19. Also, it is not easy for ordinary scientists to access such big data, and the authors of this paper, despite their best efforts, were unable to access even small data, not big data. Under these circumstances, they determined the following method and applied this method. Defining a method, a way of doing something with a definite plan, we can list our phases as follows:

1. Determination of an appropriate Pareto distribution that can explain the transmission with statistical analysis.
2. Drawing the COVID-19 transmission network using the distribution obtained in the first stage, generating random numbers.
3. Identifying super-spreaders in the transmission network by network centrality measure.

However, what is ideal for them is to identify and compare the two networks they call ‘pre-pandemic’ and ‘pandemic’ networks. If the two aforementioned networks are created and compared, then the first step of the method used in the paper will be unnecessary. Obviously, the first stage of the method used in the paper is just a facility used to overcome the difficulties in finding data.

In analysing COVID-19 outbreak, most of the times, instead of focusing on a transmission probability distribution; *R*_0_ value as an average or median have been used and super-spreaders are not taking into account. But the extreme values make a long tail for this distribution and rare infection events determine the shape of this distribution.

‘Since the *R*_0_ has a key role in measuring the transmission of diseases and is crucial in preventing epidemics, thus it is important to know which methods and formulas to apply to estimate *R*_0_ and have better performance’ [2]. But we know that different methods give different results [3] and most of the times in scientific articles which method has been used is not mentioned. Besides, sometimes *R*_0_ value is given as a median; for example, it is expressed as, ‘We estimated that the median of estimated *R*_0_ is 5.7 (95% CI of 3.8–8.9)’ [4] and this may lead us to some confusion too.

‘The emerging picture for epidemic spreading in complex networks emphasizes the role of topology in epidemic modelling’ [5]. Disease transmission networks have the motifs of transmission stories. One of the most important ways to avoid contamination is to have information about how this transmission happens.

The main purpose of this study is to develop a simple method that will make it easier for us to look at the COVID-19 issue from the network science window and focusing on the interplay between network theory, statistics and epidemiology [6]. In this simple method, first we determine a transmission probability distribution and second simulating this probability distribution we can draw a transmission graph and try to understand the process contamination using this graph.

### Super-spreaders

We want to give examples to show how the same virus can have different results in different environments. In South Korea, around 40 and Washington State more than 30 people have been infected and we should also add that there are no big differences in the dates of the events as seen from footnote [7].

Looking at the outbreaks in history, it can be seen that the phenomenon of super-spreaders is not new. ‘We examine the distribution of fatalities from major pandemics in history (spanning about 2500 years), and build a statistical picture of their tail properties. Using tools from Extreme Value Theory (EVT), we show for that the distribution of the victims of infectious diseases is extremely fat-tailed’ [8]. Susceptible hosts within a population had not equal chances of becoming infected. Although ‘it is still unclear why certain individuals infect disproportionately large numbers of secondary contacts’ [9]. If we have extreme transmitters, then ‘the practice of relying on an average *R*_0_ in dynamic disease models can obscure considerable individual variation in infectiousness’ [10].

Heterogeneities in the transmission of infectious agents are known since the end of 1990s [11]. We can define the phenomenon of super-spreaders in the framework of network science as follows: ‘The super-spreaders are the nodes in a network that can maximize their impacts on other nodes, as in the case of information spreading or virus propagation’ [12]. This definition reminds us the outlier concept of statistics. But super-spreaders are not outliers that can be discarded from analysis, ‘In this framework, super-spreading events (SSEs) are not exceptional events, but important realizations from the right-hand tail of a distribution’ [13].

One of the most important features of COVID-19 in contamination in society is the Pareto principle created by super-spreaders. Super-spreaders transmit the disease to a large number of people in an outlier-like manner, resulting in few people transmitting the disease to a large number of people, and as a result we have a transmission distribution as a power-law distribution.

Since many years there has been a debate that power-law formulations are performed better than others in infectious diseases [14], researchers are beginning to come to a consensus that coronavirus transmission more or less follows the 80/20 Pareto principle [15, 16] and estimated that 20% of cases were responsible for 80% of local transmission [17].

### Network science

Network science, initiated by the famous mathematician Leonhard Euler in 1736, came with the idea that networks were formed randomly until 2000s. However, in early 2000s, this idea was overcome and it was determined by Albert Laszlo Barabassi that the networks developed as scale-free networks and that the degree distributions of these networks obeyed the power law. Interestingly, the wealthier in the scale-free networks gets richer in connectivity, and those on social networks can be thought as a potential super-spreader if they don't follow social distancing in real life.

## Methods

In the outbreak, the number of patients' variable, in other words, the number of transmissions changes only at the discrete points of time. This means that the number of transmissions in outbreaks is discrete variables. The number of COVID-19 patients varies in many situations; how many new people are infected with the disease, how many patients have recovered and how many of them are likely to be infected or infectious again. If we were able to predict the transmission processes, there would be a deterministic approach in creating a transmission network however its impossibility is clear. Therefore, this is a stochastic process, as the number of transmission and from whom it is transmitted cannot be fully estimated or determined. Probability distributions are used in stochastic simulation models. In the simulation model, the number of transmissions, which is a discrete event, varies in the number of patients depending on time (between ‘*t*’ time point and ‘*t* + 1’ time point (s ‘*t*’ and ‘*t* + 1 may be, 7 days' or 14 days' period)) which is a change or increase is a discrete value and this change or increase is a discrete value. However, since there is no definite vaccination or herd immunity, person-to-person transmission remains a dynamic process.

In simulation models, the distribution is determined by collecting information and data about the subject studied. In light of these data, the model is established by determining the probability distributions of probabilistic (stochastic) processes. In this study, as a result of literature search, the principle that COVID-19 transmission is related to Pareto distribution and power law has been adopted and we decided the distribution to be produced in the drawing of the random transmission network, as the discrete Pareto distribution. By simulating the Pareto distribution, disease transmission data from one patient to others were randomly derived. The network drawn with the derived data is created according to the power-law distribution. Power law is independent of scale. The concept of independence from the scale indicates that the ratio and probability in small numbers such as 10 and 40 are equal to the ratio and probability in large numbers such as 1000 and 40000. In such networks, few nodes have many connections, and many nodes have few connections the ‘rich get richer’ rule (preferential attachment), remains valid in connection [18].

In this study, R open source statistics software, included the igraph package was used for the simulated data analysis and with igraph (R package for network analysis and visualisation) social network analysis is drawn and statistical comparisons with network metrics are tested. ‘It can handle large graphs very well and provides functions for generating random and regular graphs, graph visualization, centrality methods and much more’ [19].

### Simulating discrete Pareto distribution (Zipf distribution)

Zipf, Pareto and power law ‘terms are used to describe phenomena where large events are rare, but small ones quite common. For example, there are few large earthquakes but many small ones. There are a few mega-cities, but many small towns. There are few words, such as “and” and “the” that occur very frequently, but many which occur rarely’ [20]. Economists know that Wilfried Fritz Pareto observed that 20% of Italians held 80% of the country's wealth in the 19th century. Pareto principle is also known as the 80/20 rule.

If we give an example of one of the studies on the transmission distribution as power law, ‘The empirical data are highly consistent with the hypothesis that the number of reported cases are taken from a truncated power-law distribution of the form *P*(*n*) ~ *n*^−*μ*^, 1 ⩽ *n* ⩽ *n*_max_’ [16].

**Fig. 1.**
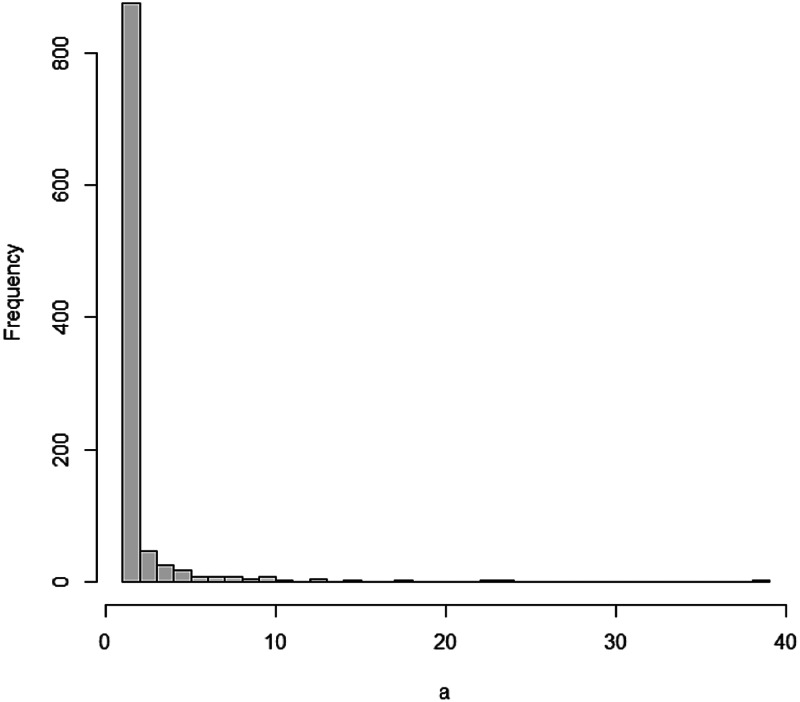
Histogram of a simulated discrete Pareto distribution.

Using the degreenet package and code in an R source [21], we can simulate a discrete Pareto distribution and draw its histogram as shown in Figure 1.

### Drawing COVID-19 transmission graphs

If all the network information is not available, then the solution for studying with large networks is to sample nodes or connections. Sampling theory for networks is similar to snowball sampling in which several nodes and their connections are sampled. In this way, information about hidden or unobservable networks behind existing nodes and connections in a small number of samples could be obtained [22]. With the discrete Pareto probability distribution, which we have theoretically justified in this paper, nodes and connections produced by simulation. Simulating nodes and connections with such sampling designs is considered a practical way of modelling the big picture.

Contact networks and disease transmission networks are different from each other. However, although data on this subject are obtained through filiation studies or contact trace apps, it is not easy to convert these data into social network-like networks due to some uncertainties and bureaucratic problems.

In this case, how to determine a synthetic COVID-19 transmission network question becomes important.

**Fig. 2.**
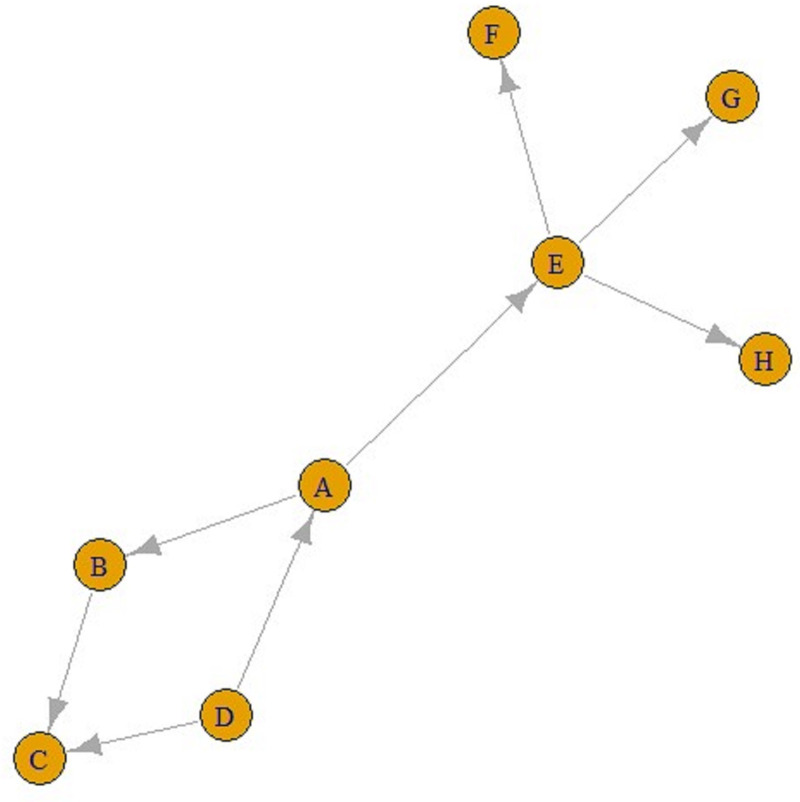
A contact graph.

The number of incoming connections in a ‘contact’ network does not have to be one. For example, in Figure 2, people B and D were placed closer than 1.5 m from C, but the people who transmitted disease to C was B, as seen in Figure 2. Briefly, nodes in contact networks can have multiple incoming and outgoing connections.

**Fig. 3.**
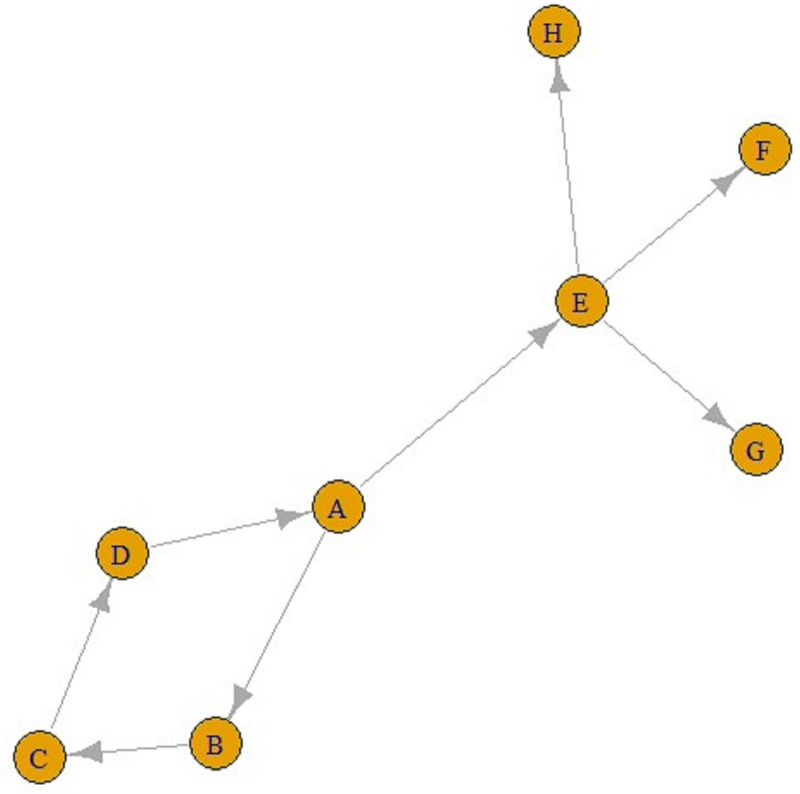
A transmission graph.

Eventually, ‘contact’ networks become ‘contamination’ networks. All these graphs come from ‘contact’ networks. The graph in Figure 2 is a ‘transmission’ graph, and as can be seen, the number of incoming connections is one for all these five nodes. In contrast, the number of outgoing connections can be an integer greater than one. For example, in Figure 3, E is infected by A and F, G and H are infected by E. Similarly, A is infected by D, and B and E are infected by A. These can be referred to as ‘transmission’ graphs.

It is also possible to find all the paths from any node to other nodes using these graphs. For example, according to the line above, there is a single ABC-shaped path leading to C in A. Similarly, from A to F can also be reached via the AEF pathway, and the number of jumps in both the ABC pathway and the AEF pathway is two.

Different personal contact network structures can reduce R, which represents how many individuals are infected by each carrier. ‘By introducing a social network approach, we propose that a decrease in R can simultaneously be achieved by managing the network structure of interpersonal contact’ [23]. If we move from this point, we can think about different network structures that will reduce infectiousness in epidemics, considering that we may encounter different outbreaks in the future.

**Fig. 4.**
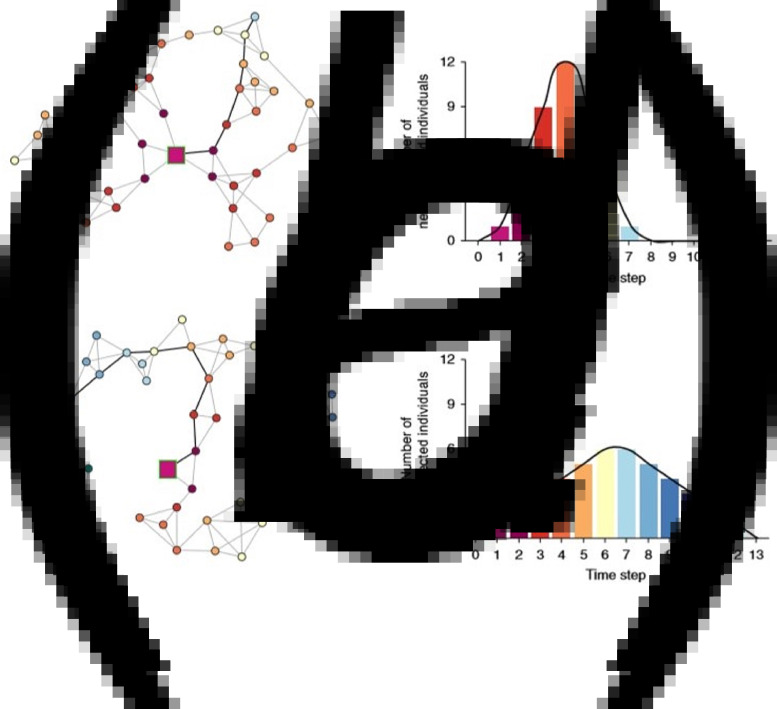
Two example networks: (a–c) with the same number of nodes and ties [23].

As it can be seen in Figure 4, ‘From a social network perspective, the shape of the infection curve is closely related to the concept of network distance (or path lengths), which indicates the number of network steps needed to connect two nodes’ [23, 24].

In Figure 4, two example networks, a–c, have the same number of nodes (individuals) and ties (social interactions) but different structures (shorter path lengths in a and longer path lengths in c), which imply different infection curves (b and d, respectively). Bold ties highlight the shortest infection path from the infection source to the last-infected individual in the respective networks. Network node colour indicates at which step a node is infected and maps onto the colours of the histogram bars [23].

In order to obtain such a network, first we need to have a probability distribution regarding the number of people a person can infect the disease. When we have such a transmission probability distribution about the probabilities that a person can transmit the disease to how many people, we can draw a social network-like COVID-19 transmission graph by generating random variables about how many people can transmit the disease at each stage.

**Fig. 5.**
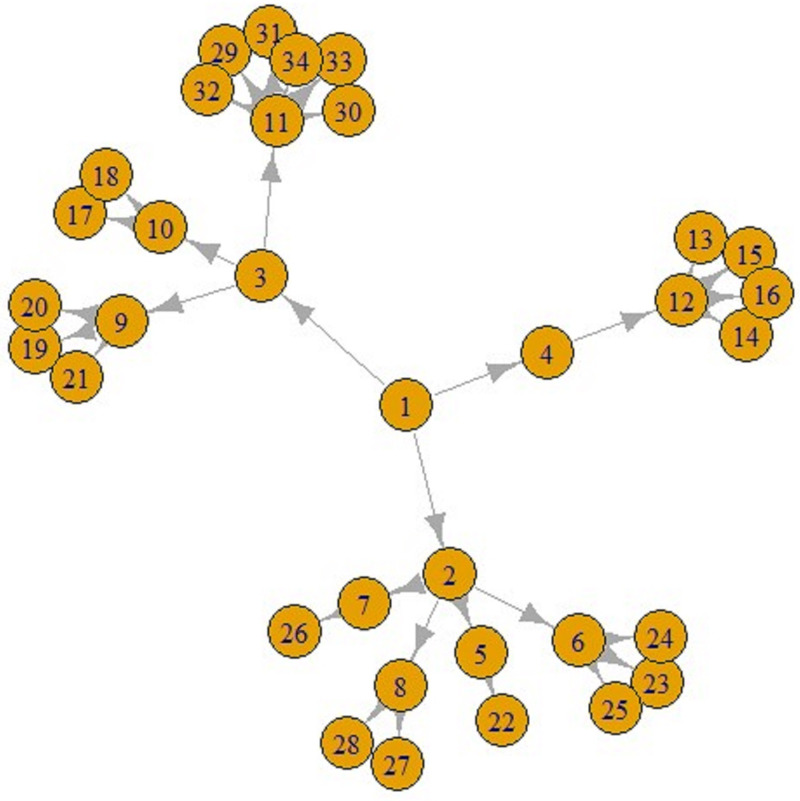
COVID-19 transmission graph using simulated discrete Pareto distribution values.

**Fig. 6.**
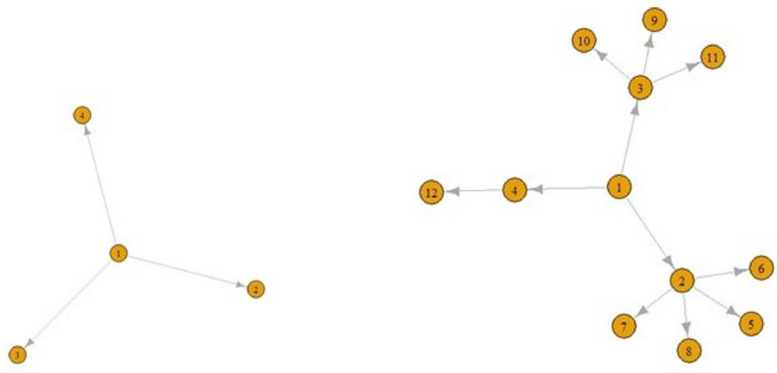
The first (left) and second stages (right) of COVID-19 transmission graphs.

Figures 5 and 6 display a COVID-19 transmission graph using simulated discrete Pareto distribution values. In Figure 6 we can see the first and second stages of these graphs. In the third stage, shape of the graph transforms into that shown in Figure 5. And we used eigenvalue centrality measure to determine which nodes are super-spreaders.

We have found that node 3 is a super-spreader, as seen in Figure 5.

## Results

This study, which is conducted within the framework of interdisciplinary approach, focuses mainly on two purposes. In the first of these purposes transforming a transmission distribution of statistics and epidemiology into a transmission network of network science is aimed.

In the second one, we try to determine who the super-spreaders are by using this network. For this step, we generated the values obtained by simulating a discrete Pareto distribution and drew and interpreted the transmission network.

The main finding of our Pareto distribution simulated data study is the similarities with a study performed using real data in India (Figs 5 and 6) [25].

## Discussion

It is not appropriate to express the transmission distribution of this disease with an average *R*_0_ because this distribution is a power law descending from left to right. But at the beginning of the COVID-19 outbreak, this mistake has been made in the analysis. However, with all the criticisms we have made, we should also point out that traditional epidemiological techniques have been used in many countries for a long time and have brought great benefits

And the second mistake is not to consider the importance of super-spreaders in this distribution. Looking at the outbreaks in history, it can be seen that the phenomenon of super-spreaders is not new and spanning about 2500 years and the distribution of the victims of infectious diseases is extremely fat-tailed. Most of the times, the fact that 80% of the infection is carried out by a 20% group is often not considered and most of the analysis begin with a *R*_0_ reproduction number. In fact, we should add that there is a connection between these two errors and that this is a single error.

The super-spreaders are the nodes in a network that can maximise their impacts on other nodes, in the case of virus transmission. Although it is still unclear why certain individuals infect disproportionately large numbers of secondary contacts, the fact that 80% of the infection is carried out by a 20% group is important. But maybe it is necessary to add that some research tells us that 80% of secondary transmissions may have been caused by a small fraction of infectious individuals (~10%).

We know that, ‘Typically the network structure is inferred from indirect, incomplete, and often biased observations. Specification of an adjacency matrix is even more difficult when the underlying network is dynamic’ and another centrality measure named expected force has been offered for additional advantages over existing spreading power and centrality measures [25].

We have to underline that point; determination of transmission probability distribution is a very important point in the analysis of the epidemic and determining the precautions to be taken. We know that in such a case network structure is inferred from indirect, incomplete, and often biased observations. As the main difficulty in establishing such a link is the lack of data and information due to the very recent COVID-19 that is why required data are generated by simulation. After transforming a transmission distribution into a transmission network and having such a graph, also we may compute many network measures and use these measures in our decision process.

Disease transmission networks have the motifs of transmission stories. One of the most important ways to avoid contamination is to have information about how this transmission happens. From a social network perspective, the shape of the infection curve is closely related to the concept of network distance (or path lengths), which indicates the number of network steps needed to connect two nodes.

Contact networks and disease transmission networks are different from each other. Incoming and outcoming edges can be any number in a contact network but in a transmission network the number of incoming edge always must be one and outcoming edge can be any number like contact networks.

In our opinion, the contribution of this paper is to provide an introduction to how the epidemiological phenomenon of identifying super-spreaders can be viewed from a network window. Undoubtedly, this paper is only an introduction to solving such problems, and the success of such an approach largely depends on the creation of the two networks we have mentioned with qualified data. And the main limitation of this research is lack of real data and inability to verify with it.

In addition, among all these super-spreader equations, it is very important to be evaluated in asymptomatic cases that change the epidemic process. In a study showing the transmission of COVID-19, it was shown that more than half of the people who tested positive were asymptomatic. The study also highlights that control strategies that focus only on symptomatic cases are insufficient to prevent contamination and understand transmission dynamics [26].

In our study, we have used eigenvalue centrality measure to determine which nodes are super-spreaders. In fact, the issue does not end at this point and is just beginning, because only if the biological, social and genetic features of the determined super-spreaders can be determined, only then can these people be prevented from accelerating the outbreak.

## Data Availability

All the data used in this study are available through the cited references and codes.

## References

[1] WHO/Europe. Coronavirus disease (COVID-19) outbreak. Available at https://www.euro.who.int/en/health-topics/health-emergencies/coronavirus-covid-19/novel-coronavirus-2019-ncov (Accessed 19 July 2020).

[2] Nikbakht R (2019) Comparison of methods to estimate basic reproduction number (*R*_0_) of influenza, using Canada 2009 and 2017–18 A (H1N1) data. Journal of Research in Medical Sciences 24, 67. Published online: 24 July 2019. doi:10.4103/jrms.JRMS_888_18. PMC6670001 31523253

[3] Breban R, Vardavas R and Blower S (2007) Theory versus data: how to calculate *R*_0_? PLoS ONE 2, e282. 17356693 10.1371/journal.pone.0000282 PMC1804098

[4] Sanche S (2020) High contagiousness and rapid spread of severe acute respiratory syndrome coronavirus 2. Emerging Infectious Diseases 26, 1470–1477. 32255761 10.3201/eid2607.200282 PMC7323562

[5] Pastor-Satorras R and Vespignani A (2001) Epidemic spreading in scale-free networks. Physical Review Letters 86, 3200–3203. 11290142 10.1103/PhysRevLett.86.3200

[6] Danon L (2011) Networks and the epidemiology of infectious disease. Interdisciplinary Perspectives on Infectious Diseases 2011, 284909. 21437001 10.1155/2011/284909 PMC3062985

[7] McGraw E. A few superspreaders transmit the majority of coronavirus cases. *The Conversation* Available at http://theconversation.com/a-few-superspreaders-transmit-the-majority-of-coronavirus-cases-139950 (Accessed 10 July 2020).

[8] Cirillo P and Taleb NN (2020) Tail risk of contagious diseases. Nature Physics 16, 606–613.

[9] Stein RA (2011) Super-spreaders in infectious diseases. International Journal of Infectious Diseases: IJID 15, e510–e513. 21737332 10.1016/j.ijid.2010.06.020 PMC7110524

[10] Sneppen K, Taylor RJ and Simonsen L (2020) Impact of Superspreaders on dissemination and mitigation of COVID-19. *medRxiv*, doi:10.1101/2020.05.17.20104745.

[11] Woolhouse ME (1997) Heterogeneities in the transmission of infectious agents: implications for the design of control programs. Proceedings of the National Academy of Sciences of the United States of America 94, 338–342. 8990210 10.1073/pnas.94.1.338 PMC19338

[12] Madotto A and Liu J (2016) Super-spreader identification using meta-centrality. Scientific Reports 6, 38994. 28008949 10.1038/srep38994 PMC5180094

[13] Lloyd-Smith JO (2005) Superspreading and the effect of individual variation on disease emergence. Nature 438, 355–359. 16292310 10.1038/nature04153 PMC7094981

[14] Meyer S and Held L (2014) Power-law models for infectious disease spread. Annals of Applied Statistics 8, 1612–1639.

[15] MIT Technology Review. What's a coronavirus superspreader? Available at https://www.technologyreview.com/2020/06/15/1003576/whats-a-coronavirus-superspreader/ (Accessed 10 July 2020).

[16] Blasius B (2020) Power-law distribution in the number of confirmed COVID-19 cases. *arXiv:2004.00940 [nlin, q-bio]*; Published online: 30 April 2020. 10.1063/5.0013031 PMC7519452 33003939

[17] Adam DC (2020) Clustering and superspreading potential of SARS-CoV-2 infections in Hong Kong. Nature Medicine. Published online: 17 September 2020. doi: 10.1038/s41591-020-1092-0. 32943787

[18] Gursakal N (2009) *Sosyal Ağ Analizi (Social Network Analysis)*. Dora.

[19] Project R (2020) *R Project for Statistical Computing, igraph: Network Analysis and Visualization*.

[20] Adamic L. *Zipf, Power-law, Pareto – a ranking tutorial. HP Labs* Available at https://www.hpl.hp.com/research/idl/papers/ranking/ranking.html (Accessed 10 July 2020).

[21] Rdrr R. Package Documentation. *simdp: Simulate from a Discrete Pareto Distribution in degreenet: Models for Skewed Count Distributions Relevant to Networks* Available at https://rdrr.io/cran/degreenet/man/simdp.html (Accessed 10 July 2020).

[22] Frank O (1978) Sampling and estimation in large social networks. Social Networks 1, 91–101.

[23] Block P (2020) Social network-based distancing strategies to flatten the COVID-19 curve in a post-lockdown world. Nature Human Behaviour 4, 588–596. 10.1038/s41562-020-0898-6 32499576

[24] Wasserman S and Faust K (1995) *Social Network Analysis Sociology: general interest*.

[25] Sahasranaman A and Kumar N (2020) Network structure of COVID-19 spread and the lacuna in India's testing strategy. *arXiv:2003.09715 [physics, q-bio]*; Published online: 21 March 2020.

[26] Arons MM, (2020) Presymptomatic SARS-CoV-2 infections and transmission in a skilled nursing facility. New England Journal of Medicine 382, 2081–2090. 32329971 10.1056/NEJMoa2008457 PMC7200056

